# Hygienic grooming is induced by contact chemicals in *Drosophila melanogaster*

**DOI:** 10.3389/fnbeh.2014.00254

**Published:** 2014-07-23

**Authors:** Aya Yanagawa, Alexandra M. A. Guigue, Frédéric Marion-Poll

**Affiliations:** ^1^Division of Creative Research and Development of Humanosphere, Research Institute for Sustainable Humanosphere, Kyoto UniversityUji, Japan; ^2^Institut National de la Recherche Agronomique, UMR iEES-ParisVersailles, France; ^3^Laboratoire Evolution, Génomes, Spéciation, Centre National de la Recherche Scientifique, UPR 9034Gif-sur-Yvette, France; ^4^Département Sciences de la Vie et Santé, AgroParisTechParis, France

**Keywords:** *Escherichia coli*, LPS, contact chemoreceptors, wing margin, grooming behavior

## Abstract

In social insects, grooming is considered as a behavioral defense against pathogen and parasite infections since it contributes to remove microbes from their cuticle. However, stimuli which trigger this behavior are not well characterized yet. We examined if activating contact chemoreceptive sensilla could trigger grooming activities in *Drosophila melanogaster*. We monitored the grooming responses of decapitated flies to compounds known to activate the immune system, e.g., dead *Escherichia coli* (Ec) and lipopolysaccharides (LPS), and to tastants such as quinine, sucrose, and salt. LPS, quinine, and Ec were quite effective in triggering grooming movements when touching the distal border of the wings and the legs, while sucrose had no effect. Contact chemoreceptors are necessary and sufficient to elicit such responses, as grooming could not be elicited by LPS in *poxn* mutants deprived of external taste sensilla, and as grooming was elicited by light when a channel rhodopsin receptor was expressed in bitter-sensitive cells expressing *Gr33a*. Contact chemoreceptors distributed along the distal border of the wings respond to these tastants by an increased spiking activity, in response to quinine, Ec, LPS, sucrose, and KCl. These results demonstrate for the first time that bacterial compounds trigger grooming activities in *D. melanogaster*, and indicate that contact chemoreceptors located on the wings participate in the detection of such chemicals.

## Introduction

Insects, especially Diptera, devote a considerable amount of time to self-grooming. Grooming involves brushing the body and the wings with the legs, and cleaning the legs and the antenna with the mouthparts. In cockroaches, grooming helps cleaning external chemosensory receptors (Böröczky et al., 2013). Stereotyped grooming occur in response to mechanical stimulation of dorsal bristles in flies (Burg et al., 1993), of eye bristles in cricket (Hensler, 1986), or of leg and wing hairs in locusts (Burrows and Newland, 1994; Newland and Burrows, 1994, 1997; Page and Matheson, 2004). Grooming or scratching is also observed in response to noxious molecules in flies (Dethier, 1972) and in locusts (Newland, 1998; Page and Matheson, 2004). Grooming thus serves a number of purposes, related to maintaining the integrity of the body and avoiding noxious stimuli.

In analogy with the documented roles of grooming in other terrestrial animals, grooming may help insects to reduce the impact of ectoparasites (Mooring et al., 2004). In social insects like ants and termites, self- and allo-grooming activities contribute to reduce the pathogenicity of bacterial diseases and entomopathogenic fungi (Boucias et al., 1996; Shimizu and Yamaji, 2002; Traniello et al., 2002; Yanagawa and Shimizu, 2007; Aubert and Richard, 2008; Yanagawa et al., 2008). Self-grooming activities may also be important for solitary insects such as flies, which live in an environment littered with microorganisms (Wölfle et al., 2009; Stensmyr et al., 2012). In line with this hypothesis, flies groom themselves to clean a dust applied to them (Phillis et al., 1993). Social situations increase the rate of grooming, especially in females (Connolly, 1968) and grooming is always performed after oviposition (Yang et al., 2008), i.e., situations with a higher risk of contamination from microbes.

If one purpose of grooming is directly linked with the need of cleaning the body from potential ectoparasites (Zhukovskaya et al., 2013), then it would make sense that grooming were triggered by signals emanating from the microorganisms. One of these stimuli is probably noxiousness (Elwood, 2011), as spores sticking to the cuticula need first to pierce it to invade the body. Volatile stimuli could also be involved. In flies, specific olfactory receptors are devoted to the detection of harmful microbes (Stensmyr et al., 2012). In termites, we have shown that odors from pathogenic spores are detected by the olfactory system (Yanagawa et al., 2009) and that such odors trigger allo-grooming (Yanagawa et al., 2010, 2011, 2012). This opens the possibility that contact chemicals related to microbes could also trigger grooming in insects.

In this work, we examined if activating contact chemoreceptors triggers grooming activities in adult flies *Drosophila melanogaster*. In order to dispose of a simple behavioral test, we used decapitated flies. The main experimental advantage of this approach, is that decapitated flies do not fly and that they stand on their legs and remain responsive for up to 20 h (Vandervorst and Ghysen, 1980). In crickets, complete grooming sequences can be executed after transection of the connectives anterior to the mesothoracic ganglion (Berkowitz and Laurent, 1996a,b). In flies, localized grooming responses to mechanical stimulation of sensilla located on different segments of the body can be observed after decapitation (Vandervorst and Ghysen, 1980). This response is considered as a simple reflex that involves a reduced circuitry, which has been studied extensively (Corfas and Dudai, 1989; Burg et al., 1993; Phillis et al., 1993; Yellman et al., 1997; Ashton et al., 2001; Kays et al., 2014).

As contact chemoreceptors are present mostly on the legs and on the wings in decapitated flies (Stocker, 1994), we stimulated these appendages with bacterial compounds, e.g., dead *Escherichia coli* (Ec) and lipopolysaccharides (LPS), and with non-volatile chemicals known to be either aversive (quinine), appetitive (sucrose) or both depending on the concentration (NaCl and KCl) (Meunier et al., 2003). We first determined which substances and doses were more effective in triggering grooming upon contact with the distal border of the wings and the legs. We then evaluated if mechanosensation alone was sufficient to elicit a response to LPS by using mutant flies deprived of external taste sensilla (Nottebohm et al., 1994). We further tested if chemosensation alone was sufficient to induce a grooming response by inducing optogenetic specific activation of taste neurons responding to bitter molecules (Nagel et al., 2003; Hornstein et al., 2009). Finally, by using electrophysiology, we tested if the contact chemoreceptors located on the margin of the distal part of the wing were sensitive to the tastants tested in this study. Our observations support the hypothesis that contact chemicals play a decisive role in triggering grooming activities in *D. melanogaster*.

## Materials and methods

### Flies

*D. melanogaster* flies were maintained on a standard cornmeal agar food at 20°C and 80% humidity. Most experiments were done with Canton Special (CS) flies. Flies devoid of external taste sensilla were Poxn^70^/Cyo; MKRS, Sb/TM6B, Tb (*Poxn*^70^).

In order to generate flies in which optogenetic activation of taste neurons responding to bitter substances was possible, we crossed flies carrying a *Gr33a-Gal4* construction (generously given by J. Carlson) with flies carrying UAS-channel rhodopsin *(UAS-CHR2)* (Bloomington Drosophila Stock Center, stock no. 28995). Since the balancer chromosome of this construction carried the defect *curly* (CyO), we could select in the progeny individuals which did not express the construction (called mutant) and others (siblings) which expressed the phenotype. For *Gr33a*, the genotype was Gr33a-Gal4^[1]^ /CyO; Dr/TM3, Sb, Ser × UAS-H134R-CHR2, where the siblings expressed the curly wing phenotype and the mutants had normal wings. During development, the larvae were fed on normal medium added with 1 mM *trans*-retinal (Hornstein et al., 2009). Adults were stimulated with blue light during 3 min, using an LED Laser at 480 nm (COO-pE-100F-WH1-20, CoolLED, UK).

### Bacteria

The TOP 10 strain of *E. coli* was incubated in liquid LB medium. *E. coli* was washed by distilled water and heated at 95°C for 5 min. From this medium, we collected a 1.4 × 10^9^/ml bacterial suspension (as measured with an absorption spectrometer). This suspension was subsequently diluted 10^0^, 10^2^, 10^4^, and 10^6^ fold.

### Chemicals

LPS (L2630, Lipopolysaccharides from *Escherichia coli* 0111:B4, Sigma), sucrose, quinine, NaCl, and KCl were provided by Sigma-Aldrich and dissolved in distilled water.

### Grooming test

All behavioral observations were made on decapitated flies: such flies were reported to remain standing and responsive to stimuli during up to 20 h (Vandervorst and Ghysen, 1980); in our experimental conditions, flies looked responsive and alive during at least 2–3 h. Flies were lightly anesthetized by placing them on ice for 3–5 min. They were then placed under a stereoscopic microscope, and 10 flies were beheaded by a single cut at the neck made with micro-scissors. Flies woke up within 2–3 min. They were placed into an upright position and allowed to recover during about 10 min. The bioassays were performed at room temperature by placing the flies on a filter paper. We used a sharpened toothpick previously soaked into the test solution, to gently touch one of their appendage (wing, foreleg, or hindleg). The subsequent grooming activities were monitored and scored up to 3 min after the stimulation. 4-day old CS flies were tested with *E. coli* in suspension into water, with LPS, sucrose, quinine, and NaCl diluted in water. *Poxn*^70^ mutants were tested with LPS. Controls were performed by stimulating flies with distilled water. Age dependent responses were examined with LPS on 1, 7, and 10-day old CS flies. Each chemical was tested on 20 female and 20 male flies.

For optogenetic experiments, flies were exposed to a continuous pulse of blue light for 3 min, over the whole body. Headless flies were placed on a filter paper as usual, but the experiment was conducted in a dark condition. The intensity of the grooming response was scaled from 0 to 5 (see table in **Figure 3B**). 4-day old flies were used for all tests.

### Visit rate test

In order to further evaluate the impact of LPS on another type of behavior, we recorded the number of flies visiting agar with or without a chemical treatment. This test monitors if flies avoid walking on a substrate or on the contrary spend more time on it (Marella et al., 2006), by counting at regular intervals how many flies are present on each substrate. Four day old flies were starved for 22 h in the presence of a wet filter paper and then transferred to cylindrical bottles (7 cm height, 3 cm diameter). The bottom was separated into two parts by mean of a strip of aluminum foil and each part was filled with 1 ml of 1% agarose and 100 mM sucrose. One side was treated with 20 μl of 10 mg/ml LPS. As a control, we proposed flies a situation where they were given the choice between agar and agar to check if their distribution was symmetrical, and between agar and agar with 100 mM sucrose to check the discriminative power of this test. Approximately 40 flies were placed in a bottle and allowed to explore the agarose for 30 min. Digital pictures of each bottle were taken every 30 s and the number of flies standing on each substrate was manually counted. A taste preference index was calculated as PI = (number flies on the test side - number flies on the water side)/(total number of flies). Data shown were obtained from 10 replicates.

### Electrophysiological recordings

Flies (4-day old) were secured to a support with tape and electrically grounded via a silver electrode contacting a drop of electrocardiogram gel (Redux^®^ Gel, Parker Laboratories Inc., USA) placed over the abdomen. Recordings were performed on the wing part near LV2 (**Figure 4A**, square a). Taste bristles of the wing margin (**Figure 4**, ▲ in **B** and arrows in **C**) were stimulated by covering their tip with a glass electrode containing an electrolyte (1 mM KCl) and the stimulus during 2 s. To avoid adaptation, consecutive stimulations were applied at least 2 min apart. The stimulus tested were *E. coli*, LPS, sucrose, quinine, and NaCl at the same concentrations as in the grooming tests (see above). As a control, we used increasing concentrations of KCl (1, 10, 100 mM and 1 M). Recordings were performed on 10 females and 10 males for each stimulus category.

The active electrode was connected to a preamplifier (TastePROBE DTP-02, Syntech) (Marion-Poll and van der Pers, 1996), and the electric signals were further amplified and filtered (CyberAmp 320, Axon Instrument, Inc., gain = 200, 8th-order Bessel pass-band filter = 1–2800 Hz). These signals were digitized (DT9803, Data Translation; sampling rate = 10 kHz, 16 bits), stored on computer, and analyzed using dbWave (Marion-Poll, 1996). Spikes were detected and analyzed using software interactive procedures of custom software dbWave. We evaluated the action-potential frequency by counting the total number of spikes during each recording.

### Statistical analysis

To examine the concentration dependent increase of the grooming behavior in headless flies with respect to sex, chemicals, and fly-strains, a multiple logistic regression (JMP 10.0 software, SAS) was applied. For the analysis of fly visits, in the 2-choices assay, a Dunnett test was applied. Optogenetic induction of grooming *via* blue light stimulation was analyzed using a Wilcoxon test. The number of spikes from electrophysiological recordings were analyzed by Wilcoxon test including sex as factor, and then the following factors: dorsal/ventral location (D/V: Table S3), sensillum number (1–5: Table S3), and sensillum types (V1, D1, V2, D2, V3, …, Table S3) for each sex.

## Results

### Grooming responses to chemical stimulation

**Figure 1:**
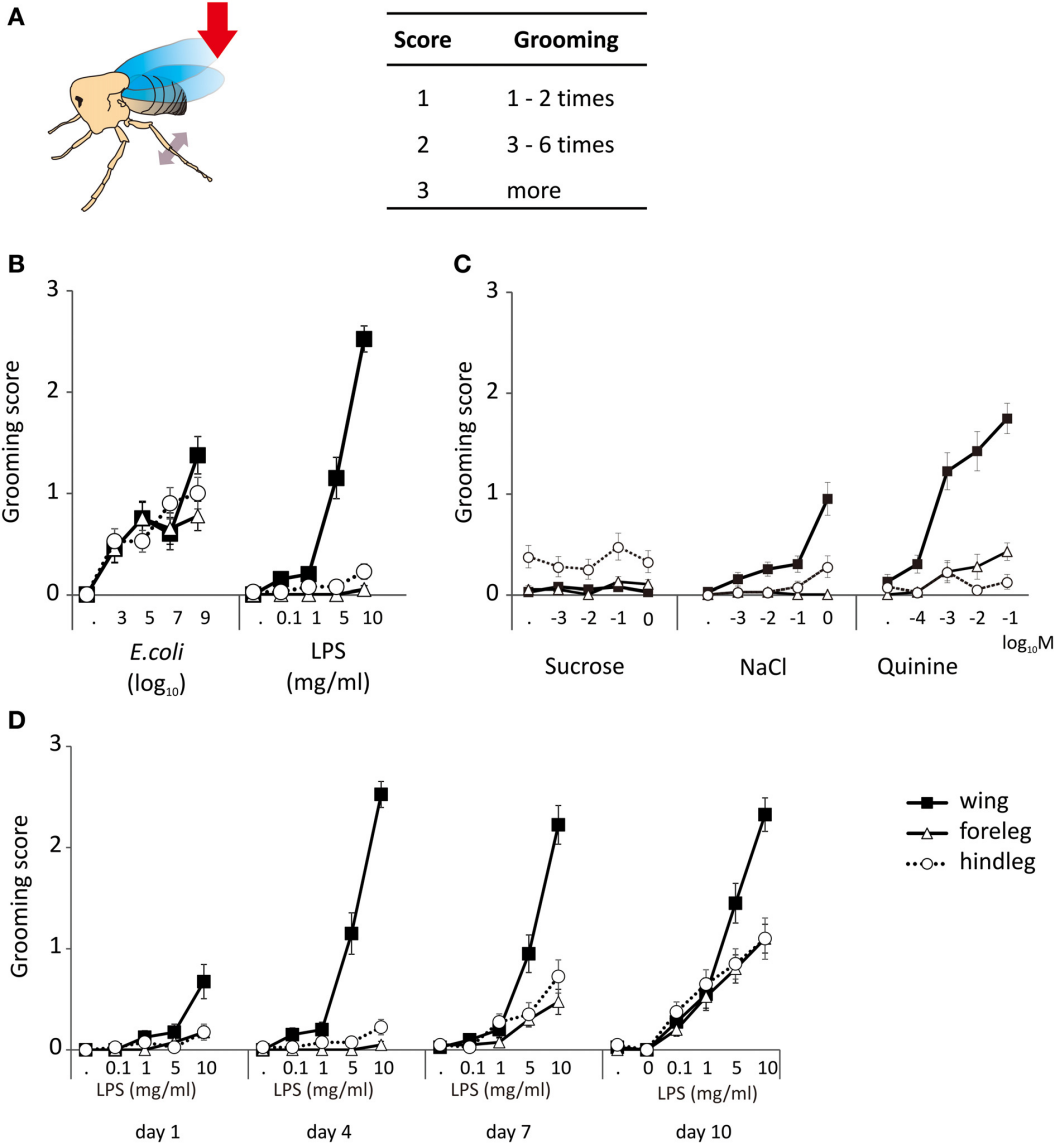
**Grooming responses. (A)** The grooming responses of decapitated flies were scored according to their intensity and duration. A stimulus is performed by touching the wing or leg with the tip of a tooth pick (red arrow). The animal responds to a proper stimulus by moving its metathoracic legs, browsing the wings or touching each other (double side arrow head). **(B)** Grooming responses induced by *E. coli* and LPS applied to the legs and the wings of 4 d old flies. **(C)** Grooming responses induced by general chemicals like sucrose, quinine, and salt. **(D)** Grooming responses to LPS appear early on the wings but develop progressively on the legs. The lowest concentration without number “·” indicated the response to water, which was used as control solution.

Decapitated flies are capable of self-grooming movements following a stimulation. In order to induce this behavior, we gently brushed their wing margin or one of their leg with the tip of a toothpick. Flies which never exhibited grooming in response to stimulation with a toothpick dipped into a chemical stimulus never showed grooming after stimulation with water alone. These movements mostly involved the meta-thoracic legs which were raised and moved independently in a succession of strokes, brushing the wings, the abdomen and the dorsum or which were extended under the abdomen and touched each other in a series of reciprocal sliding movements (Supplemental Movies 1, 2). We scored these movements according to their intensity using a scale of 0–3 according to the number of grooming sequences induced over a period of observation of 3 min (Figure 1A).

Dead *E. coli* in suspension (Ec) and LPS induced grooming responses in 4-day old adults (Figure 1B). Responses to Ec were observed following contact to the wings (*p* < 0.001, multiple logistic regression), the front legs (*p* < 0.001), and the hind legs (*p* < 0.001). Responses to LPS were obtained from the wings (*p* < 0.001) but not from the legs (front legs *p* = 0.158; hind legs *p* = 0.029).

We then asked if grooming could be triggered by stimulating the wings and the front legs with three type of tastants known to modulate feeding activities in flies, a sweet (sucrose), a bitter (quinine), and a salty one (NaCl). As in the previous experiments, water did not induce grooming responses. Also, sucrose (*p* = 0.692) did not trigger any grooming response at any concentration tested (Figure 1C). NaCl and quinine triggered a dose-dependent response when applied to the wings (*p* < 0.001). Quinine elicited a more intense grooming response than NaCl, and also triggered a dose-dependent response of the frontlegs (*p* = 0.002) but not of the hindlegs (*p* = 0.696). NaCl was not effective as quinine, but it induced a dose-dependent response from the hindleg (*p* = 0.008). The other tastants did not trigger responses when brought in contact with the legs (*p* > 0.1). There were no obvious differences between genders (Table S1).

Since all observations were made on 4-day old flies, we asked if grooming responses to LPS could change with age. As shown by Figure 1D, grooming responses to LPS applied to the different appendages increased from day 1 to day 10, developing first upon stimulation of the wings (day 4) and then being fully expressed at day 10 upon stimulation of the legs.

### Disabling contact chemoreceptors abolishes grooming responses

The previous results raise the question of which sensory modality elicited the grooming responses in the previous experiments. As chemoreceptive sensilla usually contain not only chemoreceptive neurons but also a mechanoreceptive neuron, it may be argued that the mechanosensory contact of the toothpick was a fundamental factor underlying responses recorded. Note, however, that if this were the case, no dose-response effect should have been seen in the previous experiments (Figure 1). In order to determine if the mechanosensory stimulation provided by the toothpick contact was sufficient to trigger grooming responses, we used *Poxn*^70^ flies, which are deprived of external taste chemoreceptors and present only mechanoreceptive sensilla (Nottebohm et al., 1994). We stimulated these flies with LPS which was the stimulus most effective for triggering grooming in the previous experiments (see Figure 1).

**Figure 2:**
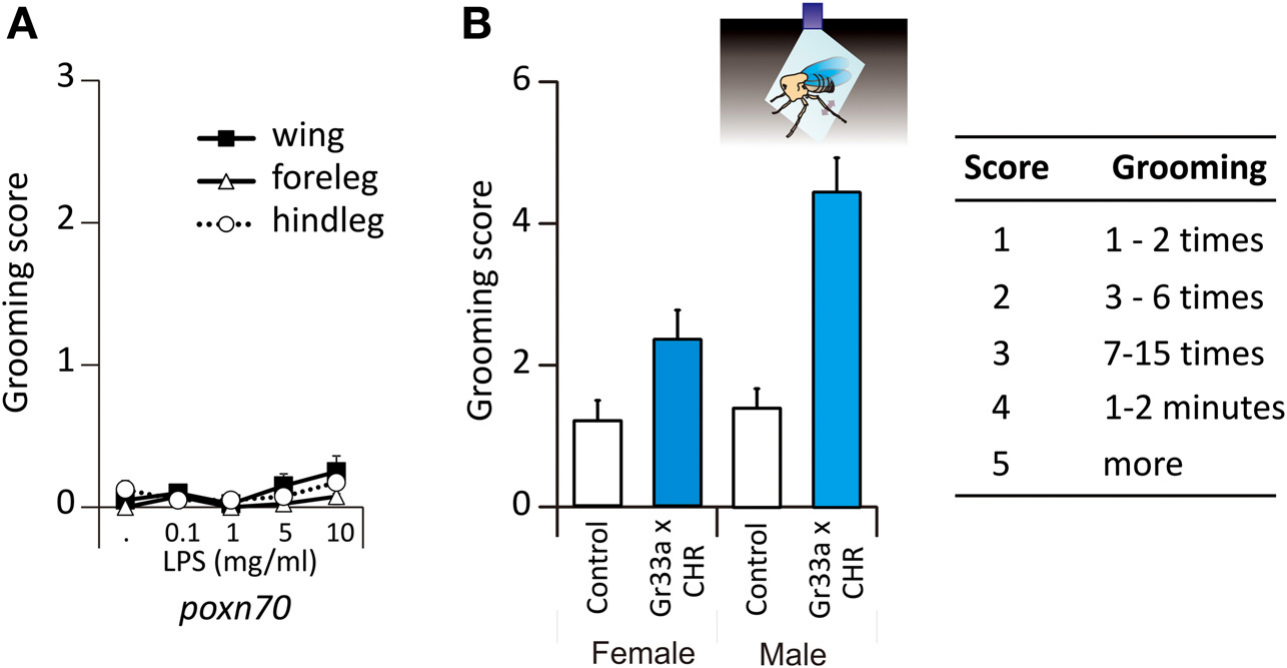
**Grooming responses to LPS in mutant flies. (A)** Grooming responses of mutant flies deprived of their taste receptors, *Poxn*^70^. **(B)** Grooming responses in bitter taste-evoked flies, *Gr33a*-Gal4 x UAS-*ChR2*. Cartoon depicting a beheaded fly illuminated with blue light. When stimulated by blue light, the fly will move its metathoracic legs as if it was a chemical stimulation (double side arrow head).

These flies did not respond to LPS following stimulation of the wings (*P* = 0.235) or of the legs (forelegs: *p* = 0.371 and hindlegs: *p* = 0.401, *n* = 40, Figure 2A). These results thus show that mechanosensory input alone cannot induce grooming so that responses to Ec, LPS, and tastants such as quinine or NaCl had a chemoreceptive basis mediated by external contact chemoreceptive sensilla.

### Stimulating contact chemoreceptors is sufficient to induce grooming

In order to bypass stimulation of the mechanoreceptors, we expressed a channel rhodopsin receptor into taste neurons in order to activate them by light only. To this end, we used the ubiquitous UAS-Gal4 system (Brand and Perrimon, 1993) to express channelrhodopsin2 (ChR2) (Nagel et al., 2003; Hornstein et al., 2009) into cells expressing *Gr33a*, which encodes for a receptor essential for aversive taste in *Drosophila* (Moon et al., 2009). As stated in the Materials and Methods section, flies carrying the construction had straight wings (mutant flies). Those which carried half of the construction exhibited curled wing tips (sibling flies) and were used as control flies. Headless flies were stimulated with a pulse of 480 nm light during 3 min. Blue light did not affect control flies (Figure 2B). In *Gr33a*-CHR2 flies, photoactivation of *Gr33a* neurons induced grooming both in males (*p* < 0.001, Wilcoxon test) and females (*p* = 0.044) in the absence of any chemosensory stimulus. We found a sexual dimorphism in the flies carrying the construction as photoactivation induced more grooming in males than in females (*p* = 0.004 in *Gr33a*-Gal4 × UAS-ChR2, Figure 2B, Table S2), but not in control flies where the response was absent (Figure 2B).

### LPS is deterrent to free-moving flies

**Figure 3:**
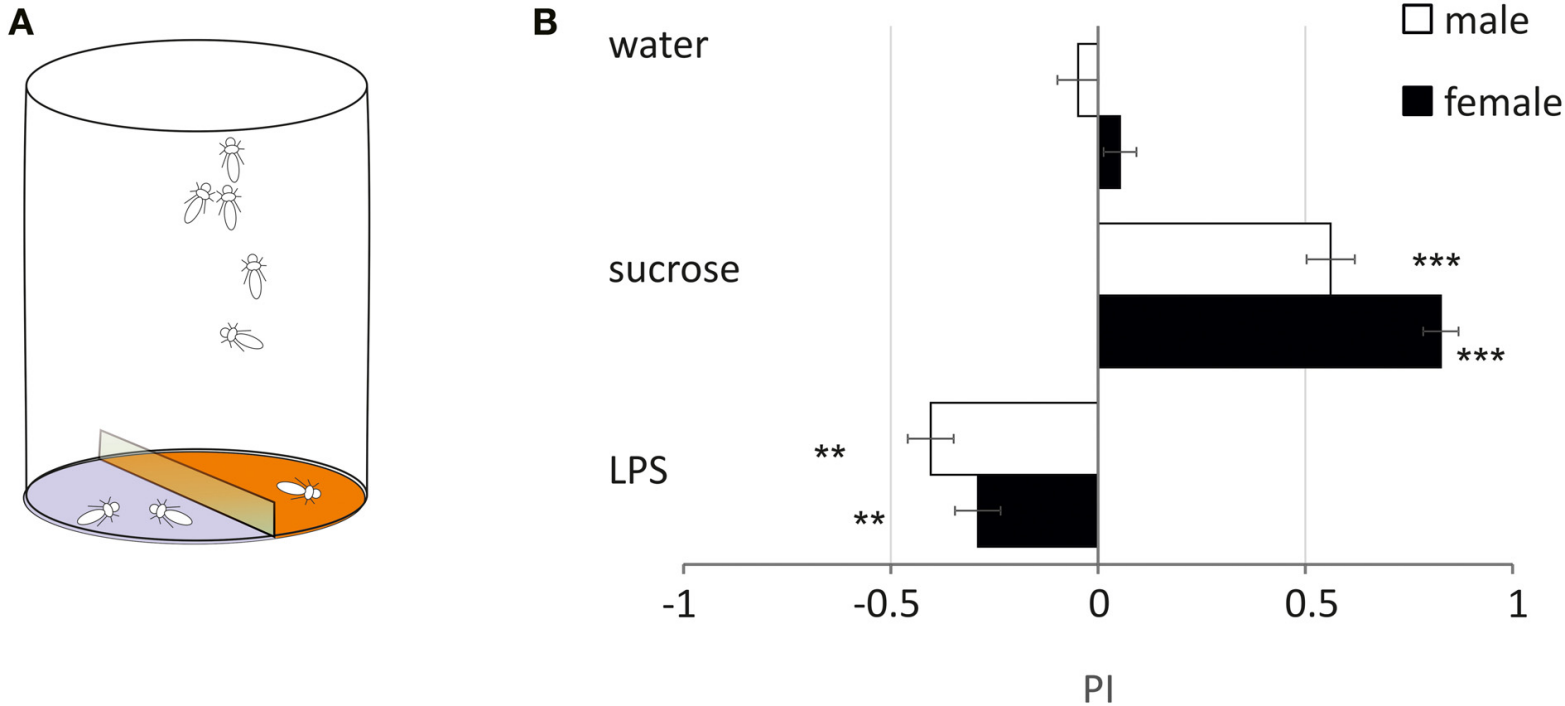
**Two-choice test. (A)** Schematics of the behavioral test. **(B)** The preferences for either side were examined by computing a preference index (P.I.) (^**^p < 0.01, ^***^p < 0.001, ANOVA). Black bars: males; white bars: females (mean ± s.e.m., *n* = 10).

In order to evaluate if LPS triggers only grooming or if it has other influence on the walking behavior of flies, we monitored the number of flies walking over 2 agar substrates, one of which was treated (Figure 3A). With pure agar, flies visited equally the 2 sides (Figure 3B). With sucrose, flies strongly preferred sucrose over agar (PI: females = 0.83 ± 0.06: *p* < 0.001, males = 0.56 ± 0.08: *p* < 0.001, Dunnett test). With LPS, flies avoided walking on side treated with LPS (females = −0.29 ± 0.08, *p* = 0.006; males = −0.36 ± 0.08, *p* = 0.049) (Figure 3). This suggests that LPS is detected by taste neurons located on the legs (and possibly on the proboscis), which mediate coordinated responses leading to the avoidance of areas where LPS is found.

### Electrophysiological recordings

**Figure 4:**
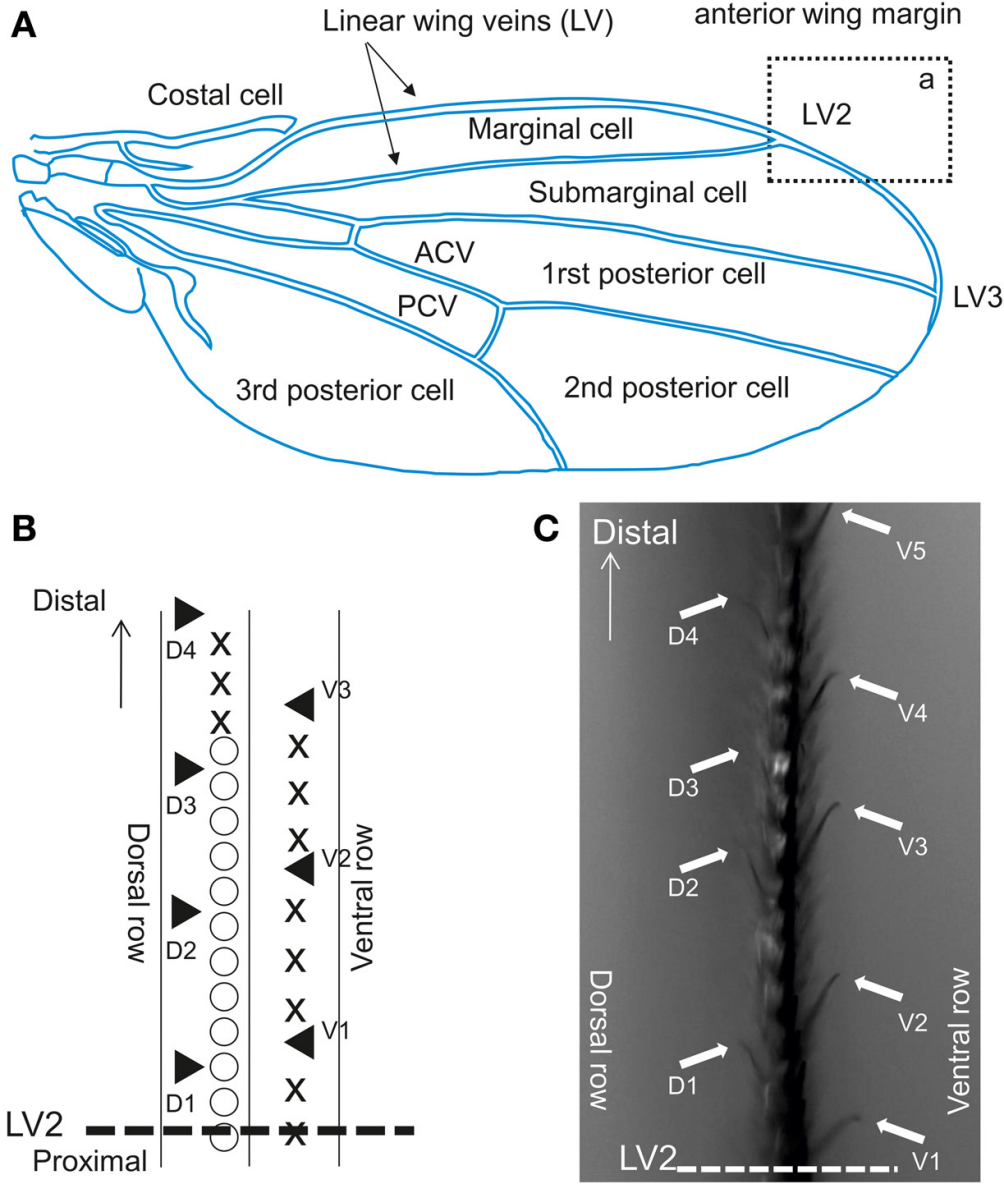
**Contact chemoreceptors on the wing. (A)** Drosophila wing. ACV, anterior cross vein; PCV, posterior cross vein. The section from which recordings were performed is outlined by a rectangle (a), which displayed at a higher magnification in Figure 2B. **(B)** Anterior wing margin, with 3 rows of bristles. Symbols on the picture show: ◯, singly innervated stout bristle; X, singly innervated slender bristle; ▲, multiple innervated curved bristles (from which recordings were obtained). **(C)** Sensilla on the vein area between LV2 and LV3 in rectangle a. Arrows indicate sensilla recorded in this study (▲ in **B**).

We investigated sensilla distributed on the anterior wing margin, located over the marginal, and sub-marginal cells across LV2 (Figure 4A). In this area, contact chemoreceptive sensilla are interspersed between stout bristles and are organized along two rows (Figure 4B), pointing upward (dorsal sensilla) or downward (ventral) (Figure 4C). Contact chemoreceptors are easily spotted under the microscope because of their curved and slender appearance, and also because their tip stands out of other bristles (see Palka et al., 1979; Isono and Morita, 2010, Figure 5). This area was selected based on pilot tests which showed that flies were more responsive when they were contacted there as compared to other regions of the wing.

**Figure 5:**
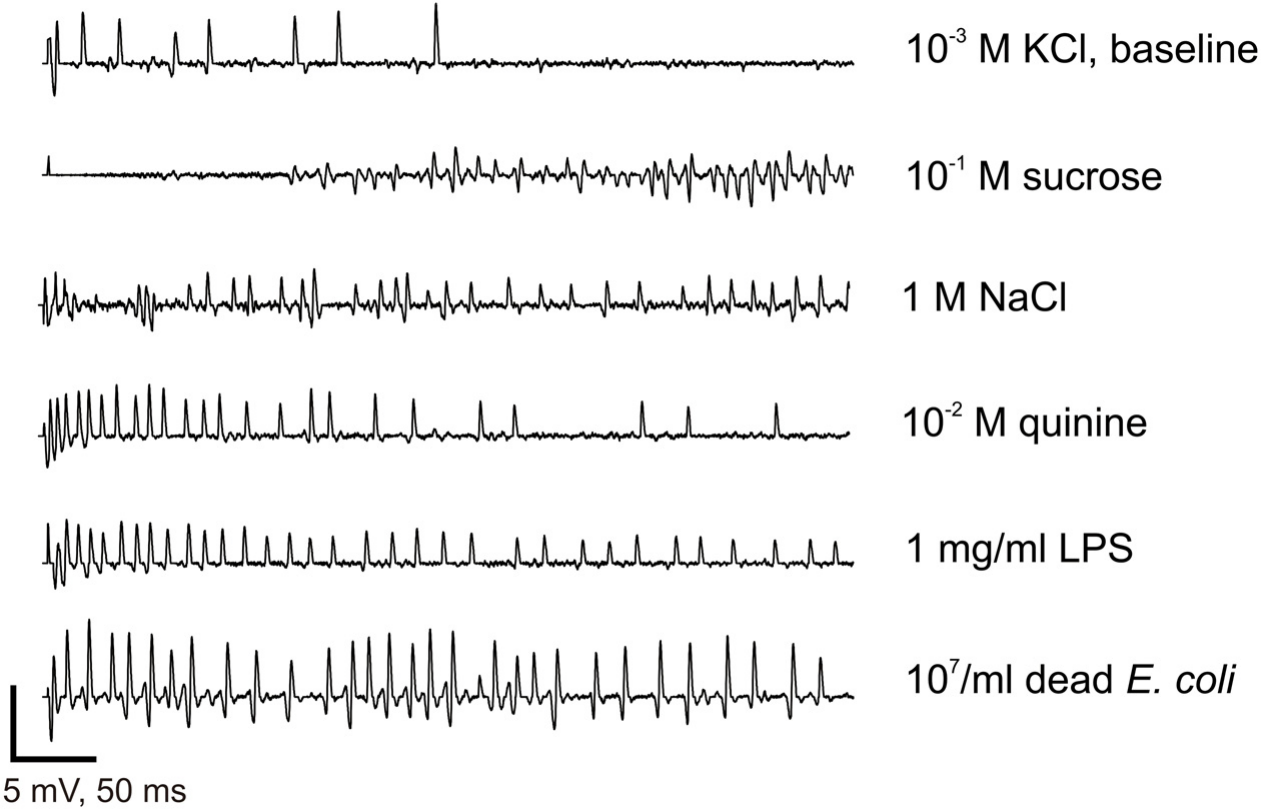
**Electrophysiological recordings from contact chemoreceptors on the wings**. Recording samples obtained from wing taste sensilla to 1 mM KCl (used as an electrolyte in all recordings), 1 M NaCl, 10 mM Quinine, 0.1 M Sucrose, 1 mg/ml LPS, and 10^7^/ml of *E. coli*. Bars on the bottom of the graph represent a scale (vertical: 5 mV, horizontal: 50 ms). Recordings were all taken from female flies, except the response to KCl.

We sampled sensilla labeled D1–D5 and V1–V5 (Figure 4). Between 30 and 50% of the sensilla were not responsive or no electrical contact could be established when contacting their tip. These sensilla are relatively slender and tend to vibrate upon contact with the stimulus electrode, much more than taste sensilla on the proboscis or on the legs. Upon contact, these sensilla occasionally responded with a phasic burst of spikes (Figure 5), possibly fired from several nerve cells.

**Figure 6:**
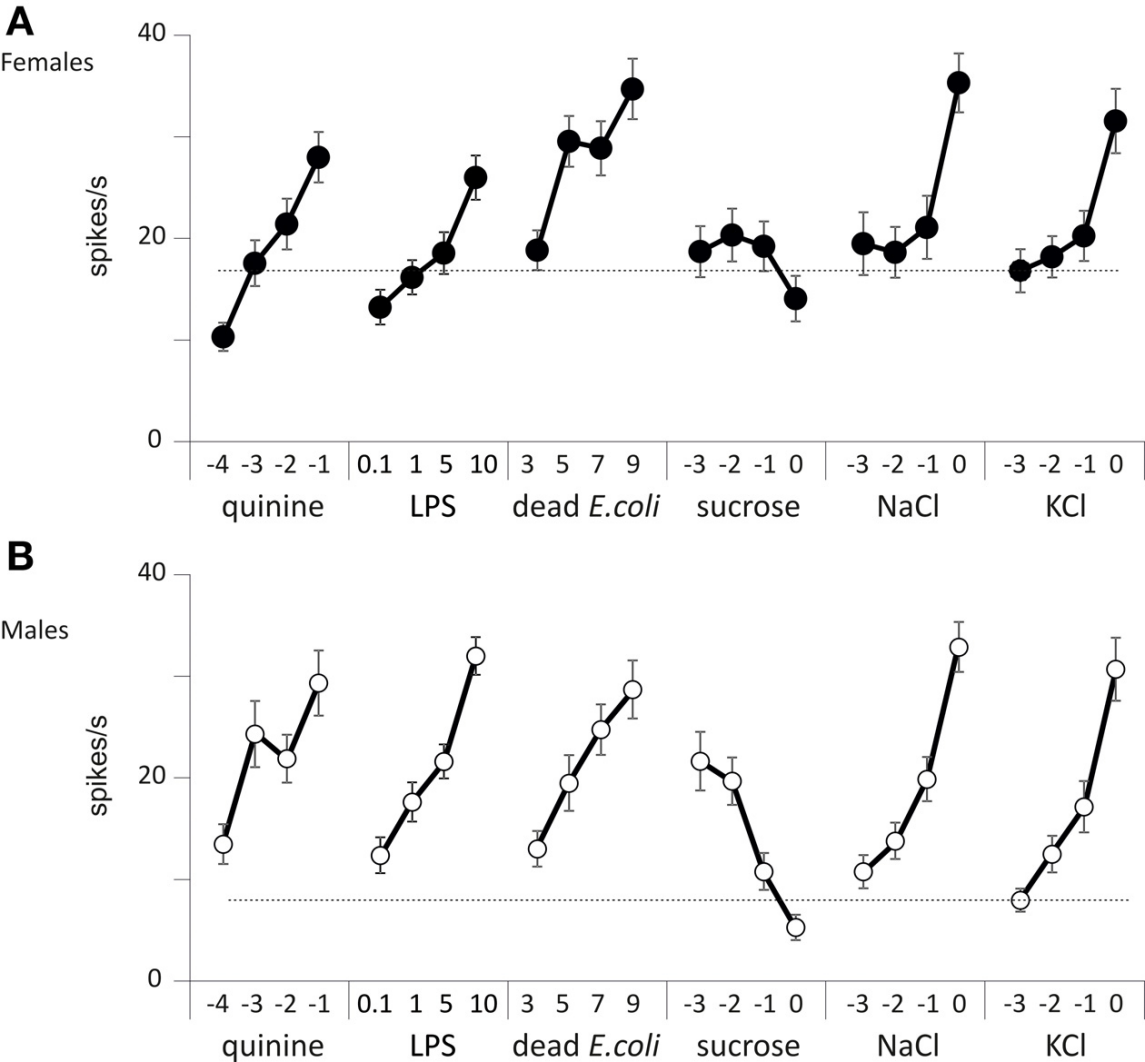
**Responses of wing taste sensilla to different chemicals**. Spiking activity recorded from wing taste sensilla in males and females in response to KCl, NaCl, quinine, sucrose, *E. coli*, and LPS. **(A)** Responses of female flies. **(B)** Responses of male flies. Each point represents the average ± s.e.m. from *n* = 51 to 89 recordings obtained from a total of 10 insects. The concentration of each stimulus is reported on the abscissa as the log_10_ of the molar concentration for KCl, NaCl, quinine, and sucrose, as log_10_ of the density per ml for dead *E. coli* and as mg/ml for LPS.

We thus counted the total number of spikes occurring within the first 2 s of the stimulation (Figure 6). When analyzing these responses over the whole set of data, we found that the number of spikes changed according to the chemical stimulus (*p* < 0.001, Wilcoxon test), to the concentration (*p* < 0.001), to sex (*p* < 0.001), and according to the sensillum position in the row (*p* < 0.001, V1–V5 and D1–D5). However, we did not find any difference between dorsal and ventral sensilla (*p* = 0.329, D/V). These observations confirm that wing contact chemoreceptive sensilla are functional, that they respond to classical taste stimuli. These sensilla show dose-dependent responses to LPS and *E. coli* suspensions. They also respond in a dose-dependent way to NaCl, to KCl, to quinine, but apparently not to sugar.

We further analyzed the temporal course of the responses by computing the number of spikes occurring during consecutive 100 ms bins (Figures S1, S2). At the lowest concentration tested, we observed a burst of spikes starting from about 20 spikes/s and decaying rapidly within 1 s. This initial response was probably elicited by the mechanoreceptor associated with these taste sensilla (in males and females, bin: *p* < 0.001 for all chemicals except KCl, *p* = 0.002 in males and *p* < 0.001 in females and NaCl, *p* = 0.254 in males and *p* = 0.420 in females, analysis of covariance). However, with sucrose, the initial burst peaked at 30–35 spikes/s which may indicate that 1 mM sucrose already elicits a response. As the concentration of each stimulus increased, the firing rate globally increased. For salts, it was mostly the tonic part of the response which was affected, while with quinine, *E. coli* and LPS both the phasic and tonic parts of the responses increased with the dose tested. On the contrary, the spiking activities were markedly depressed in both sexes when the concentration of sucrose was raised from 1 mM to 1 M.

## Discussion

We demonstrate for the first time that grooming activities in *D. melanogaster* adults are triggered by bacterial suspensions of *E. coli*, its surface compound, LPS, and by aversive chemicals like quinine and NaCl at a high concentration. Mechanosensation is not required to elicit this response as sucrose and water did not induce grooming, and *Poxn*^70^ mutants deprived of their external taste receptors were insensitive to LPS. Furthermore, grooming activities could be elicited without physical contact with a substrate by optogenetic activation of cells expressing the bitter receptor *Gr33a*. Since our behavioral observations indicated that grooming reactions were elicited by stimulating the costal outer vein of the wings, we examined the contact chemoreceptors located there using tip-recording. These sensilla were found to house contact chemoreceptive neurons increasing their firing activity in response bacterial extracts, LPS, quinine, and to high salt concentrations thus supporting the hypothesis that these wing contact chemoreceptors contribute to triggering grooming responses in decapitated flies.

In this work, we recorded grooming by scoring the intensity of the behavior in relation to the number of strokes made by the legs and by the duration of this reaction. A more detailed analysis of this behavior was not relevant here as we focused on finding chemical stimuli which could trigger this behavior. In order to simplify the experimental procedures, we also observed the reactions of decapitated insects. In such insects, the responses induced bypass the downstream control normally exerted by higher order nervous centers in intact animals. Our observations are thus by no mean representative of the normal grooming behavior but they give us a good indication of the strength of the sensory stimuli sent to the local ganglia.

While self-grooming has been described to occur in response to touch (Page and Matheson, 2004) and by noxious chemicals (Newland, 1998; Elwood, 2011), or spontaneously (Yellman et al., 1997), we demonstrate for the first time that grooming is elicited by bacterial chemicals (LPS, Ec). Since it is often said that insects use behavioral resistance to complement their immune reactions (Hughes et al., 2002), detecting LPS and bacterial-related compounds may contribute to help insects avoiding pathogens, by triggering hygienic behaviors like grooming.

LPS is not only triggering grooming responses, but it is also a deterrent as flies avoided to walk on a substrate treated with LPS (Figure 3). LPS could represent a specific avoidance signals as these molecules are present in the cell wall of all Gram-negative bacteria. However, LPS are complex molecules consisting of a lipid anchor, a polysaccharide core, and chains of carbohydrates (Salton and Kim, 1996). It remains to be seen if flies are capable of detecting such large and complex molecules either directly or following a degradation inside of the sensillum lymph. LPS may also act indirectly on the physiology of the taste neurons by virtue of its structure which resembles a surfactant. Alternately, as commercial sources of LPS are extracted from bacteria, flies could be sensitive to contaminants of LPS, such as endotoxins proteins (Hirschfeld et al., 2000) or peptidoglycans (MacKenzie et al., 2010), which trigger immune reactions in vertebrates more efficiently than LPS.

The chemicals contained in dead *E. coli* bacteria suspension are a mixture of proteins, nucleic acids and to a lesser extent lipids and polysaccharides, and the recognition and behavioral induction cascade can be much more complex. Like LPS, this mixture is a powerful stimulant for the immune system of flies (Lemaitre and Hoffmann, 2007). However, it can also be a signal for food source or for oviposition since *E. coli* easily stays on meats or ripen fruits (Van Elsas et al., 2011; Nguyen-The, 2012). Thus chemical signals from *E. coli* and LPS could work differently in hygiene behavior by the interaction with other behaviors like feeding.

Our work stresses the need to further examine the role wing contact chemoreceptors. According to Stocker (1994), each wing of *D. melanogaster* is equipped with about 40 taste sensilla, each of which hosts 4 chemosensitive neurons and 1 mechanoreceptor. Unexpectedly, we found that contact chemicals delivered specifically to the wings were quite effective in triggering grooming, and that the response to LPS was established earlier on the wings than on the legs. This role of wing contact chemoreceptors in *Drosophila* contrasts the lesser role they play in locusts, where it was found that stimulating leg contact chemoreceptors was much more effective in triggering a cleaning reaction than by stimulating their wing contact chemoreceptors (Page and Matheson, 2004).

To our knowledge, electrophysiological responses from wing contact chemoreceptors have been recorded in very few insects and the role of these chemosensitive sensilla is not clear. Wolbarsht and Dethier (1958), stressed that in the blowfly, *Phormia regina*, these sensilla respond to salts and sugar, and are quite sensitive to mechanical vibrations, suggesting that one of their function is to provide proprioceptive information during flight. However, they also reported that the axons projecting to the central nervous system are very thin which suggests that their conduction velocity is slow, making their role in controlling flight unlikely. Angioy et al. (1981a,b) suggested that wing contact chemoreceptors in *P. regina* are involved in feeding. They observed that proboscis extension and feeding attempts could be triggered by touching single wing hairs with sugar and that salt inhibits proboscis extension. In tse-tse flies, Deportes et al. (1994) examined wing contact chemoreceptors on the costal vein and recorded electrophysiological responses to mechanical stimulation and to salt, sugar, and extracts from conspecifics. They suggested that wing contact chemoreceptors play a role in social or sexual interactions. In the locust, Page and Matheson (2004) recorded responses of basiconica sensilla to noxious stimuli, which were thus considered more as playing a role in proprioception. Our observations suggest that contact chemoreceptors of the wings might play a decisive role in signaling the presence of potentially harmful microbes. Recently, Raad (2013) remarked that contact chemoreceptive sensilla are located mainly on the costal vein of the wings, precisely where a vortex occurs during wings flapping. This lead him to propose that flying insects could use their wing taste sensilla to monitor molecules trapped within this vortex, and thus detect chemicals from a flower for example, without actually touching them.

Except in the locust (Page and Matheson, 2004), all attempts to record electrophysiological responses from wing contact chemoreceptors reported a high number of unresponsive sensilla (Dethier, 1972; Angioy et al., 1979, 1981a,b; Deportes et al., 1994). Our study makes no exception. We were unable to record responses from other parts than the outer margin of the wing, although nothing distinguishes these hairs from the others from a morphological point of view. In our hands, only about 40% of the hairs of the outer margin were responsive which is much less than on the proboscis (Hiroi et al., 2002) or on the legs (Meunier et al., 2003), where the rate of success was closer to 80–90%. Deportes et al. (1994) reported similar figures with regions of the wing where no successful recordings could be obtained and with proportions of unresponsive hairs depending on the stimulus used. Angioy et al. (1981a,b) also reported unusual proportions of unresponsive hairs in *Phormia*. They suggested that unresponsiveness could be related to satiety as these contact chemoreceptors increased their resistance markedly after forced-feeding (Angioy et al., 1979). At this stage, we have no explanation for this and further experiments are warranted to determine if contact chemoreceptors of the wings are less functional than taste sensilla located on other appendages.

Raad (2013) introduced a functional technique to evaluate the sensitivity of wing chemosensitive sensilla which may alleviate the problems faced by extracellular electrophysiology. In *Drosophila*, using a Gal4 driver strongly expressed in neurons (tubulin) to drive the expression of a calcium-sensitive probe, G-cAMP (Nakai et al., 2001), he showed well-defined temporal changes of calcium concentrations following stimulation with sugars and bitter compounds in contact chemoreceptive sensilla located in the proximal part of the costal vein of the wing. He further reported that contact chemoreceptive sensilla of this area are expressing a range of sugar- and bitter-sensitive gustatory receptors of the *Gr* family, thus confirming that contact chemoreceptive sensilla of the wings of *Drosophila* are fully functional and respond at least to some sapid molecules detected by contact chemoreceptors located on the legs and on the proboscis.

In our tests, sucrose is the only stimulus which did not elicit grooming in our panel of stimuli. The electrophysiological responses obtained from wing taste sensilla did not show any increase of the firing activity with the dose, but on the contrary revealed an inhibition of the basal firing activity. This observation is consistent with recent results obtained by Raad (2013) on fly wing contact chemoreceptors using calcium imaging which failed to reveal any response to sucrose, while strong activities were found in response to glucose, fructose, and quinine. Therefore, the most likely explanation is that wing taste chemoreceptors are not sensitive to sucrose. That sucrose would inhibit the firing activity of taste neurons in a dose-dependent way is unprecedented to our knowledge, but it could be a new case of inhibitory interactions between tastants (Meunier et al., 2003; Cocco and Glendinning, 2012; Charlu et al., 2013; Jeong et al., 2013). Further observations are warranted to confirm this observation and to clarify which mechanism is at work.

So far, our findings support that contact chemicals play a decisive role in triggering hygiene grooming in *D. melanogaster*. Our study shows that LPS induces vigorous grooming activities from flies and also elicits consistent spiking activities in contact chemoreceptors of the wing. LPS has been used repeatedly in multiple organisms as an inducer of the immune system. LPS and bacterial peptidoglycans are strong inducers of the immune system in D*rosophila* (Charroux et al., 2009). Bacterial peptidoglycans are recognized by peptidoglycan recognition proteins, which can be soluble or membrane-bound, and activate both Toll and Imd pathways (Gottar et al., 2006; Gendrin et al., 2009).

### Conflict of interest statement

The authors declare that the research was conducted in the absence of any commercial or financial relationships that could be construed as a potential conflict of interest.
