# Supplementary material for: Hygienic grooming is induced by contact chemicals in Drosophila melanogaster
Source: Front Behav Neurosci. 2014 Jul 23;8:254. doi: 10.3389/fnbeh.2014.00254 (PMC4107972; doi:10.3389/fnbeh.2014.00254)
Supplement: Supplementary file 1 [file DataSheet1.DOCX]

**Supplementary data**

**
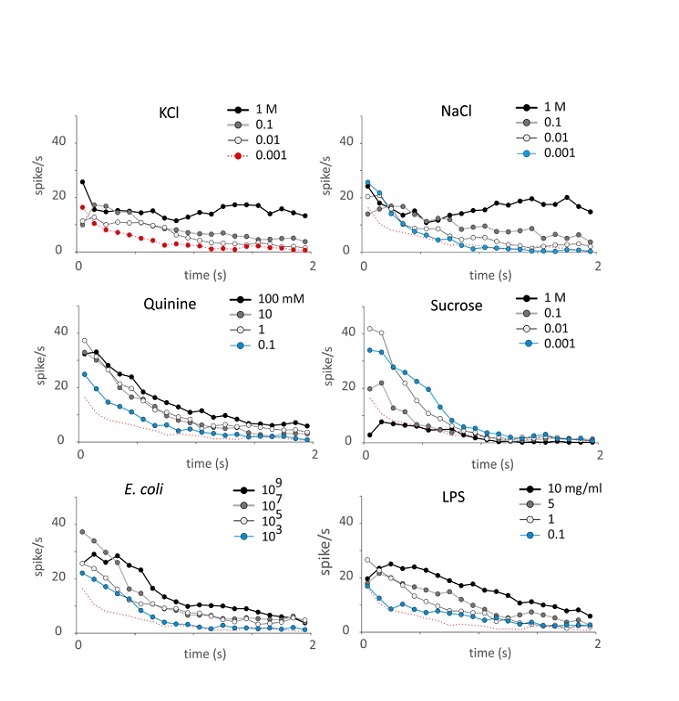
Figure S1: PSTH of the responses recorded in male wing taste sensilla**

Post-stimulus histogram (PSTH) of the responses obtained by counting the total number of spikes recorded within consecutive 100 ms bins, in response to increasing concentrations of a stimulus. Each point represents the average of the responses from 51 to 89 recordings obtained on n=10 insects. The lowest concentration is displayed with a blue line and blue circles, while the highest concentration is displayed in filled black circles connected with a heavy black bar. 2 intermediary concentrations were recorded and are displayed on the same graph with a light grey circle and a black circle. The concentrations ranges are KCl, NaCl, Sucrose: 1 mM to 1 M step log_10_ M, Quinine: 0.1 mM to 100 mM step log_10_ M, *E. coli*: 10^3^ to 10^9^ particles / ml step 2 log_10_ and LPS 0.1, 1, 5 and 10 mg/ml.


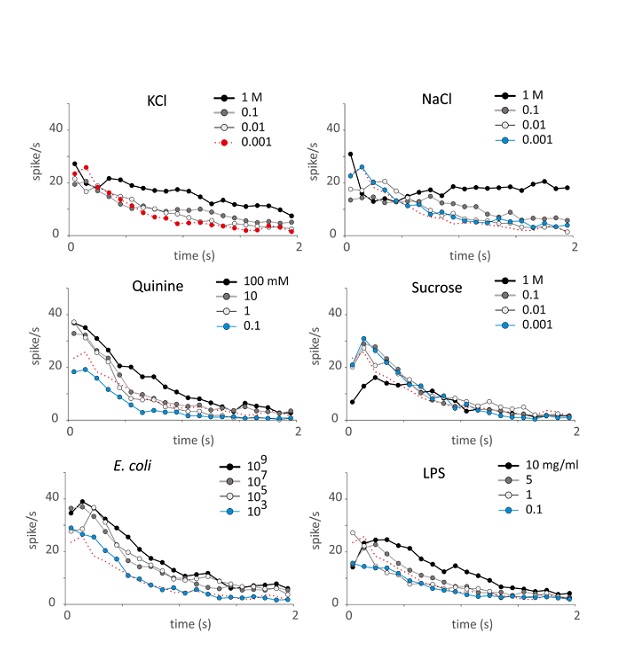
**Figure S2: PSTHs of the responses recorded in female wing taste sensilla**

PSTHs of responses recorded from females (n=53 to 87 recordings per point). Same legend as Figure S2.

**Supplemental movie 1**

grooming induction by 10^-1^ M quinine, which showed score 2 grooming

**Supplemental movie 2**

no-grooming induction by water, which showed score 0 grooming.

**Table S1. Logistic regression analysis of the grooming responses induced by contact chemicals**

Results of a logistic regression.

| **P value** | parameter | |  | parameter | |  | parameter | |
| --- | --- | --- | --- | --- | --- | --- | --- | --- |
|  | Conc. | Sex |  | Conc. | Sex |  | Conc. | Sex |
| **4-day-old flies** | | | | | | | | |
| ***E. coli*** | | | **LPS** | | |  |  |  |
| wing | p<0.001 | p=0.532 | wing | p<0.001 | p=0.016 |  |  |  |
| foreleg | p<0.001 | p=0.719 | foreleg | p=0.087 | p=0.152 |  |  |  |
| hindleg | p<0.001 | p=0.050 | hindleg | p=0.038 | p=0.127 |  |  |  |
|  | | | | | | | | |
| **Effect of age on the response to LPS** | | | | | | | | |
| 1-day | | | 7-day | | | 10-day | | |
| wing | p<0.001 | p=0.705 | wing | p<0.001 | p=0.376 | wing | p<0.001 | p=0.301 |
| foreleg | p<0.001 | p=0.007 | foreleg | p<0.001 | p=0.528 | foreleg | p<0.001 | p=0.917 |
| hindleg | p=0.004 | p=0.007 | hindleg | p<0.001 | p=0.528 | hindleg | p<0.001 | p=0.147 |
|  | | | | | | | | |
| **4 day-old flies** | | | | | | | | |
| **Sucrose** | | | **NaCl** | | | **Quinine** | | |
| wing | p=0.717 | p=0.053 | wing | p<0.001 | p=0.705 | wing | p<0.001 | p=0.705 |
| foreleg | p=0.181 | p=0.151 | foreleg | p=0.562 | p=1 | foreleg | p=0.002 | p=0.792 |
| hindleg | p=0.277 | p=0.892 | foreleg | p=0.008 | p=0.369 | foreleg | p=0.696 | p=0.375 |

n = 40 for the analysis of concentration-depending increase (Conc.: 20 females + 20 males) and n = 20 for the analysis of sex-dependent difference (Sex: 20 individuals for each sex).

**Table S2: Behavioral responses observed with *Poxn^70^* and *Gr33a*-ChR2 flies.**

Results of a logistic regression.

| **P value** | Parameter |  |
| --- | --- | --- |
|  | |  |
| Poxn^70^ to LPS | Conc. | Sex |
| foreleg | p=0.153 | p=0.154 |
| hindleg | p=0.371 | p=0.128 |
| wing | p=0.401 | p=0.148 |
|  |  |  |
| *Gr33a* x ChR2 | Induction |  |
| Female | P=0.044 |  |
| Male | p<0.001 |  |

n = 40 for the analysis of concentration-dependent difference (Conc.: 20 females + 20 males) and n = 20 for the analysis of sex-dependent difference (Sex: 20 individuals for each sex).

**Table S3: Wilcoxon test analysis of the electrophysiological responses from taste sensilla of the wings.**

Results of Wilcox test on number of spikes for each parameter.

|  | Parameter | | | | |
| --- | --- | --- | --- | --- | --- |
| stimulus | conc. | sex | D/V | location | type |
| KCl | p < 0.001 | p = 0.015 | p = 0.361 | p < 0.001 | p = 0.002 |
| NaCl | p < 0.001 | p = 0.307 | p = 0.053 | p = 0.566 | p = 0.083 |
| Quinine | p < 0.001 | p = 0.457 | p = 0.792 | p = 0.051 | p = 0.271 |
| Sucrose | p < 0.001 | p < 0.001 | p = 0.031 | p = 0.030 | p = 0.040 |
| *E. coli* | p < 0.001 | p < 0.001 | p = 0.102 | p = 0.028 | p = 0.075 |
| LPS | p < 0.001 | p = 0.029 | p = 0.129 | p = 0.027 | p = 0.011 |

Parameters tested:

- conc: stimulus concentration
- sex: gender
- D/V: dorsal *versus* ventral side of the wing
- location: positional index of the sensillum from LV3 (1-5)
- type: sensillum ID (D1, D2 …V4, V5).
